# Supplementary material for: Activin B Regulates Fibroblasts to Promote Granulation Tissue Formation and Angiogenesis During Murine Skin-Wound Healing via the JNK/ERK Signaling Pathway
Source: Int J Mol Sci. 2025 Oct 22;26(21):10284. doi: 10.3390/ijms262110284 (PMC12607779; doi:10.3390/ijms262110284)
Supplement: Supplementary file 1 [file ijms-26-10284-s001.zip › ijms-3899688-supplementary.pdf]

Activin B regulates fibroblasts to promote granulation tissue formation and angiogenesis during murine skin-wound healing via the JNK/ERK signaling pathway

Supporting Information

Supplementary Figure (Figure S)

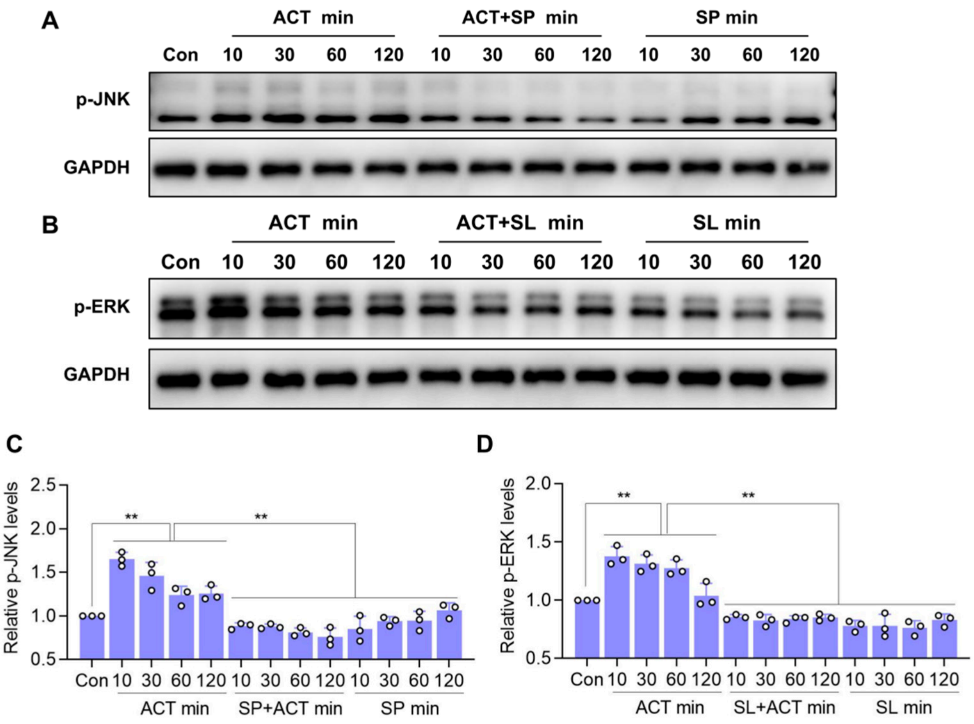

Figure S1. SP600125 and SL-327 significantly reduced ACT-induced phosphorylation levels of JNK and ERK respectively. (a,b) Western blot analysis of p-JNK, p-ERK, and GAPDH in the MAPK signaling pathway of HDFs. (c,d) Quantification of the relative phosphorylation levels of p-JNK and p-ERK, p-p38, n=3. Con: Control group; ACT: Activin B group; SP: SP600125; SL: SL327; $^{**}p < 0.01$,

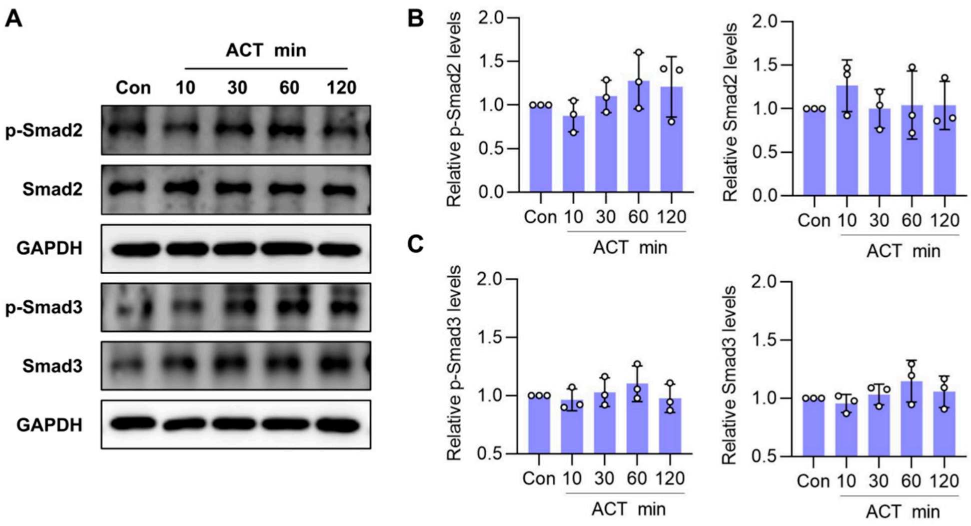

Figure S2. 10ng/ml of activin B does not significantly induce the phosphorylation of smad2/3 in human dermal fibroblasts. (a) Western blot analysis of smad2, smad3,and their phosphorylation levels, and GAPDH of HDFs. (b-c) Quantification of the relative levels of p-smad2, smad2, p-smad3, and smad3, n=3. Con: Control group; ACT: Activin B group;
